# Gut Microbiota Dysbiosis in Systemic Lupus Erythematosus: Novel Insights into Mechanisms and Promising Therapeutic Strategies

**DOI:** 10.3389/fimmu.2021.799788

**Published:** 2021-12-03

**Authors:** Quanren Pan, Fengbiao Guo, Yanyan Huang, Aifen Li, Shuxian Chen, Jiaxuan Chen, Hua-feng Liu, Qingjun Pan

**Affiliations:** Key Laboratory of Prevention and Management of Chronic Kidney Disease of Zhanjiang City, Institute of Nephrology, Affiliated Hospital of Guangdong Medical University, Zhanjiang, China

**Keywords:** systemic lupus erythematosus, autoimmune disease, gut microbiota dysbiosis, extracellular vesicle, miRNA, mesenchymal stem cell therapy

## Abstract

Systemic lupus erythematosus (SLE) is a chronic autoimmune disease that was traditionally thought to be closely related to genetic and environmental risk factors. Although treatment options for SLE with hormones, immunosuppressants, and biologic drugs are now available, the rates of clinical response and functional remission of these drugs are still not satisfactory. Currently, emerging evidence suggests that gut microbiota dysbiosis may play crucial roles in the occurrence and development of SLE, and manipulation of targeting the gut microbiota holds great promises for the successful treatment of SLE. The possible mechanisms of gut microbiota dysbiosis in SLE have not yet been well identified to date, although they may include molecular mimicry, impaired intestinal barrier function and leaky gut, bacterial biofilms, intestinal specific pathogen infection, gender bias, intestinal epithelial cells autophagy, and extracellular vesicles and microRNAs. Potential therapies for modulating gut microbiota in SLE include oral antibiotic therapy, fecal microbiota transplantation, glucocorticoid therapy, regulation of intestinal epithelial cells autophagy, extracellular vesicle-derived miRNA therapy, mesenchymal stem cell therapy, and vaccination. This review summarizes novel insights into the mechanisms of microbiota dysbiosis in SLE and promising therapeutic strategies, which may help improve our understanding of the pathogenesis of SLE and provide novel therapies for SLE.

## 1 Introduction

Systemic lupus erythematosus (SLE) is a chronic autoimmune disease characterized by the generation of autoantibodies and immune complexes, which can cause multiple organ damage to the skin, kidney, and central nervous system (1). The pathogenesis of SLE is very complex and is traditionally thought to be closely related to genetic and environmental risk factors (2). Infection is a significant cause of morbidity, disease exacerbation, and death in patients with SLE (3, 4). Recently, increasing evidence has shown that different degrees of intestinal-infection-related dysbacteriosis exist in patients with SLE and SLE mice, which are closely related to the development of SLE (5, 6). Previous studies have shown that the mechanisms associating gut microbiota dysbiosis and SLE pathogenesis include immune system imbalance, molecular mimicry, impaired intestinal barrier function, biofilms, and sex hormones. Under normal circumstances, the special barrier functions of the intestine include physical, biochemical, and immune barriers, which can separate the host from the environment. Intestinal epithelial cells are joined by tight junction proteins to form the intestinal physical barrier (7). In patients with SLE, impaired intestinal barrier function leads to increased intestinal permeability, allowing pathogens, toxins, and bacteria to leak out of the gut lumen and translocate to other organs, which is called a “leaky gut” (8). In addition, the antigens of the translocated bacteria are similar to some of the host’s structures, which cause cross-reactivity to produce autoantibodies and damage target organs in patients with SLE, a process called molecular mimicry (9, 10). Furthermore, curli amyloid in biofilms is associated with autoantibody production. Gut microbiota dysbiosis in SLE is sex-biased, which may be due to sex hormones (11).

The treatment of SLE mainly includes immune regulation and immunosuppression, with the aim of maintaining long-term remission or low disease activity, protecting organ function, and avoiding complications and adverse drug reactions (12, 13). Currently, although treatment options for SLE with hormones, immunosuppressants, and biologic drugs are now available, the rates of the clinical response and functional remission of these drugs are still not satisfactory, which may lead to serious side effects (14, 15). Therefore, there is an urgent need to develop treatment options that have good therapeutic effects in patients with few adverse effects. Emerging evidence has shown that intestinal dysbacteriosis may play an essential role in the pathogenesis of SLE and may be a novel therapeutic target for SLE. Previous studies have shown that interventions targeting the gut microbiota for SLE include dietary interventions, probiotics or prebiotics, antibiotic therapy, vaccination, and fecal microbiota transplantation (FMT). These treatments are currently only studied in lupus murine models, and further clinical trials are required to confirm their efficacy.

**Figure 1:**
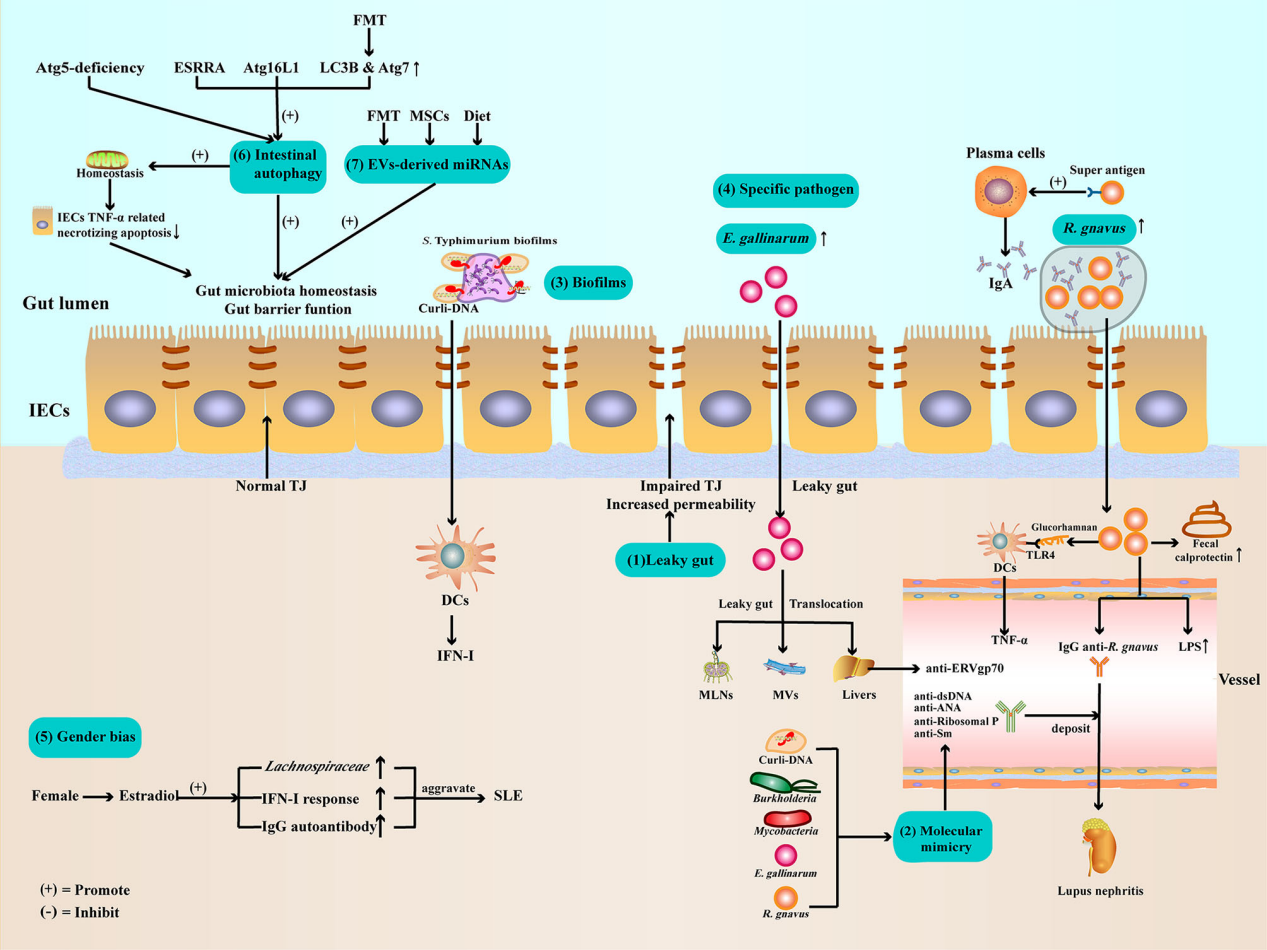
Potential mechanisms of gut microbiota dysbiosis in SLE (1). Gut barrier function impaired and leaky gut allow pathogen leak out of the gut lumen and translocate to other organs (2). Gut microbiota and Curli-DNA of biofilms produce autoantibodies through molecular mimicry, which deposit in kidneys, leading to lupus nephritis (3). Curli-DNA of biofilms activated DCs to secrete pathogenic IFN-I (4). *E. gallinarum* can disrupt *intestinal barrier* function and translocate to MLNs, MVs, and livers. At the same time, *E. gallinarum* promoted systemic autoimmunity by inducing ERV gp70 overexpression in the liver. *R. gnavus* express a B-cell superantigen to stimulate IgA antibodies production and encapsulate itself to facilitate intestinal colonization. Furthermore, *R. gnavus* can produce a glucorhamnan inflammatory polysaccharide that promotes DCs to secrete the inflammatory factor TNF-α *via* TLR4. In addition, *R. gnavus* can disrupt intestinal barrier function, resulting in increased calprotectin levels in stool samples and LPS levels in sera. Subsequently, the impaired intestinal barrier function exposes the intestinal commensal *R. gnavus* antigen, leading to mimicry of the molecule to produce anti-dsDNA autoantibodies, aggravating lupus nephritis (5). Estradiol promotes pathogen like *Lachnospiraceae* colonization, IFN-I response, and IgG autoantibody production (6). Regulate ESRRA, Atg16L1, LC3B, and Atg7 can activate IECs autophagy to maintenance gut microbiota homeostasis and intestinal barrier function (7). Evs-derived miRNAs from FMT, MSCs therapy, or dietary improve gut microbiota balance and enhance intestinal barrier function. ATG, autophagy-related protein; DCs, dendritic cells; *E. gallinarum*, *Enterococcus gallinarum*; ESRRA, estrogen related receptor alpha; FMT, Fecal microbiota transplantation; IECs, intestinal epithelial cells; IFN-I, type I interferon; LC3B, microtubule-associated protein 1 light chain 3B; LPSs, lipopolysaccharides; MLNs, mesenteric lymph nodes; MVs, Mesenteric veins; MSCs, mesenchymal stem cells; *R. gnavus*, *Ruminococcus gnavus*; TJ, tight junction; TLR4, toll-like receptor4; TNF-α, Tumor necrosis factor-α.

**Figure 2:**
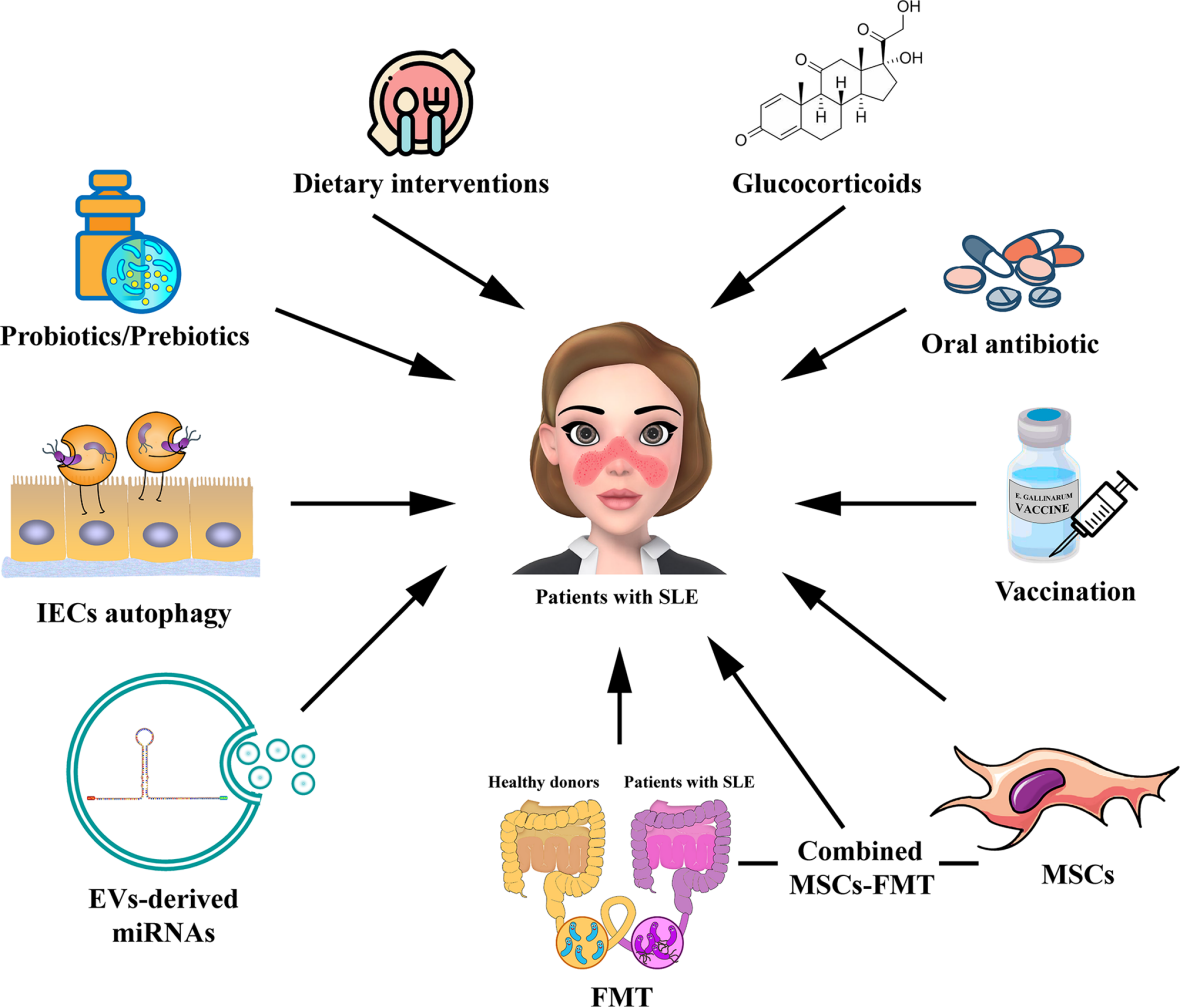
Potential strategies for targeting gut microbiota in the treatment of patients with SLE. The potential therapies for modulating gut microbiota for SLE, including probiotic or prebiotic therapy, dietary interventions, oral antibiotic therapy, GC therapy, vaccination, FMT, regulation of IECs autophagy, EV-derived miRNA therapy, and MSC therapy. The combined MSC-FMT transplantation approach may have a better therapeutic effect for SLE. EV, extracellular vesicle; GC, glucocorticoid; FMT, Fecal microbiota transplantation; MSCs, mesenchymal stem cells.

In this review, we summarize the gut microbiota dysbiosis in patients with SLE and mouse models, and first described the possible role of IECs autophagy, and extracellular vesicles (EVs) and miRNA, in the gut microbiota homeostasis of SLE, as shown in **Figure 1**. In addition, we propose several novel treatment strategies targeting gut microbiota, including regulation of IECs autophagy, EV-derived miRNA therapy, and mesenchymal stem cell therapy, which may have great value for SLE treatment in the future, as shown in **Figure 2**.

## 2 Gut Microbiota Dysbiosis in Patients With SLE

Recently, many studies have attempted to determine the correlation between gut microbiota dysbiosis and SLE pathogenesis, as shown in **Table 1**. A study showed that compared with healthy controls, patients with SLE suffered from intestinal dysbiosis and had a significantly lower ratio of *Firmicutes*/*Bacteroidetes* (F/B) (22). This result was confirmed by subsequent studies (18, 19, 23, 24). Importantly, *Firmicutes* are inversely correlated with the SLE disease activity index (SLEDAI score) (20), indicating that *Firmicutes* can delay lupus progression. It follows that the reduced F/B ratio is an important manifestation of gut microbiota dysbiosis in patients with SLE. A recent study analyzed stool samples from 117 untreated patients with SLE and reported that the gut microbiota of patients with SLE showed a pro-inflammatory and autoimmune profile compared to healthy controls (6). Furthermore, patients with SLE mostly show decreased richness and diversity of intestinal microbiota compared to healthy controls (6, 8, 25), and this was particularly severe in patients with high SLEDAI scores (8).

**Table 1:** Gut microbiota dysbiosis in patients with SLE.

Study (Year)	Subjects(n)	Gut microbiota in SLE	Role of microbiota	Reference
López, et al. (2016)	SLE (20) HC (20)	**Phyla**: *Firmicutes*, *Synergistetes*↓.	*Firmicute*s was a negative correlation with Th17 cells; *Synergistetes* was a negative correlation with anti-dsDNA antibodies;	(16)
Azzouz, et al. (2019)	SLE (61) HC (17)	**Genus:** *Ruminococcus gnavus*↑.	Anti-*R. gnavus* antibodies were positively correlated with SLEDAI score, anti-dsDNA antibodies and lupus nephritis.	(8)
Bellocchi, et al. (2019)	SLE (27) HC (27)	**Genus**: *Bifidobacterium*, *Ruminiclostridium, Streptococcus* and *Collinsella*↑; *Lachnoclostridium*, *Lachnospira*, and *Sutterella* ↓.	*Streptococcus* has been associated with inflammatory intestinal conditions.	(17)
Li et al. (2019)	SLE (40) HC (20)	**Phyla**: *Firmicutes*/*Bacteroidetes* ratio↓; **Family**: *Streptococcaceae*, *Lactobacillaceae*↑ **Genus**: *Faecalibacterium*, *Roseburia*↓; *Streptococcus*, *Lactobacillus* and *Megasphaera*↑; **Species**: *F. prausnitzii*↓; *S. anginosus, L. mucosae*↑	*Streptococcus*, *Campylobacter*, *V eillonella*, *anginosus* and *dispar* were positively correlated with lupus activity; *Bifidobacterium* was negatively associated with SLE disease activity.	(18)
Guo, et al. (2020)	SLE (20) HC (20)	**Phyla**: *Firmicutes*/*Bacteroidetes* ratio↓; *Bacteroidetes*↑; **Genus**: *Dialister*, *Gemmicer*↓	*Dialister and Gemmiger* were a negative association with inflammatory cytokines.	(19)
He, et al. (2020)	SLE (21) HC (10)	**Phyla**: *Bacteroidetes*↓, *Proteobacteria*↑; **Family**: *Ruminococcaceae*↓, *Enterococcaceae*↑; **Genus**: *Clostridia* and *Faecalibacterium*↓, *Escherichia_Shigella*↑.	Most of the bacteria that are negatively correlated with SLEDAI belong to *Firmicutes*.	(20)
Chen, et al. (2021)	SLE (117) HC (115)	**Species**: *Clostridium* sp. *ATCC BAA-442*, *Atopobium rimae*, *Shuttleworthia satelles*, *Actinomyces massiliensis*, *Bacteroides fragilis*, *Clostridium leptum*↑ and	These species were reduced after treatment.	(6)
Wen, et al. (2021)	SLE (33) HC (28)	**Phyla**: *Proteobacteria* **Order**: *Enterobacteriales* **Family**: *Ruminococcaceae*		(21)

Interestingly, the abundance of *Ruminococcus gnavus* (*R. gnavus*) was elevated 5-fold in the gut microbiota of 61 patients with SLE compared to that in healthy control and was strongly associated with SLE disease activity (8). And serum anti-*R. gnavus* antibodies were positively correlated with the SLEDAI score and anti-dsDNA levels (8).

Collectively, gut microbiota dysbiosis in patients with SLE typically displays a decreased F/B ratio, richness, and diversity. Meanwhile, impaired intestinal barrier function leads to microbiome translocation, which exacerbates disease progression in patients with lupus. Furthermore, proliferation of some specific microbiota such as *R. gnavus* may be significantly related to lupus progression.

## 3 Gut Microbiota Dysbiosis in Lupus Mouse Model

Recently, many studies have also revealed gut microbiota dysbiosis in lupus mouse models, as shown in **Table 2**. Zhang et al. (26) revealed that lactobacilli significantly reduced and *Lachnospiraceae* increased in the gut microbiota of MRL/lpr mice, which was more severe in female mice. Moreover, *Lachnospiraceae* is strongly associated with lupus progression in MRL/lpr mice. In contrast, the intestinal colonization of *Lactobacillaceae* was negatively correlated to the lupus activity in mice. Those results suggested that *Lactobacillaceae* may be a probiotic in the treatment of SLE. Another study reported similar results; antibiotic treatment can eliminate harmful microbiota *Lachnospiraceae* and enrich the probiotic *Lactobacillus Spp.*, thereby attenuating lupus (27). On the other hand, many studies have found that leaky gut occurs in lupus-prone mice with impaired intestinal barrier function, resulting in increased microbial translocation, endotoxemia, and lupus progression, which can be reversed after treatment of lupus-prone mice (11, 28, 29, 34). These results suggested that impaired gut barrier function significantly influenced lupus progression.

**Table 2:** Gut microbiota dysbiosis in lupus mouse models.

Study (Year)	Mouse model	Gut microbiota in SLE	Intervention	Reference
Zhang, et al. (2014)	MRL/lpr mice *vs.* MRL/MpJ mice; B6/lpr mice *vs.* C56BL/6 mice	**Family**: *Lactobacillaceae*↓; *Lachnospiraceae*↑ *Lachnospiraceae* was associated with lupus severity. *Lactobacillaceae* was negatively correlate with lupus activity.	Retinoic acid as a dietary intervention increased lactobacilli and relieved lupus severity.	(26)
Mu, et al. (2017)	MRL/lpr mice	**Family**: *Lactobacillaceae*↓; *Lachnospiraceae*↑	Oral antibiotics therapy ameliorated lupus in MRL/lpr mice by removing *Lachnospiraceae* and enriching *Lactobacillus spp*	(27)
Manfredo Vieira, et al. (2018)	(NZW x BXSB) F1 mice *vs.* C56BL/6 mice	**Species:** *Enterococcus gallinarum*↑ *E. gallinarum* can induce intestinal barrier impairments and lupus nephritis.	Vancomycin or vaccination therapy can remove *E. gallinarum* and thus ameliorate lupus.	(28)
Mu, et al. (2019)	MRL/lpr mice *vs.* MRL/MpJ mice;	**Species:** *Lactobacillus animalis*↑ (After vancomycin treatment)	Vancomycin treatment increased *L. animalis* and ameliorated lupus symptoms in common MRL/lpr mice, but aggravated lupus in pregnant and postpartum (PP) mice.	(29)
He, et al. (2019)	MRL/lpr mice	**Genus**: *Mucispirillum*, *Oscillospira*, *Bilophila and Rikenella*↓, *Anaerostipes*↑ (After prednisone treatment)	Prednisone treatment decrease *Mucispirillum* and increase *Anaerostipe*s. Bromofuranone did not alleviate lupus but enhanced the efficacy of prednisone in the treatment of SLE.	(30)
Zhang, et al. (2020)	MRL/lpr mice *vs.* C57BL/6 mice	**Genus**: *Proteus*, *Klebsiella*, *Bilophila*, *Allobaculum*, *Bifidobacterium* and *Adlercreutzia*↓ (After prednisone treatment)	Short-term and early-stage antibiotic treatment aggravated SLE, while FMT treatment shown to be beneficial. However, short-term premorbid antibiotic treatment or FMT could inhibit the therapeutic effect of prednisone on lupus in MRL/lpr mice aged 9 to 13 weeks.	(31)
de la Visitación, et al. (2021)	NZBWF1 mice *vs.* NZW/LacJ mice	**Phyla**: *Verrucomicrobia*, *Proteobacteria*, *Bacteroidetes* and *Proteobacteria*↑; *Firmicutes*↓; **Genus**: *Parabacteroides*, *Pedobacte*r, *Olivibacter* and *Clostridium*↑	Antibiotic treatments restored the composition of gut microbiota, and inhibited the increment of blood pressure, renal injury and disease activity in lupus-prone mice.	(32)
Wang, et al. (2021)	MRL/lpr mice	**Genus**: *Ruminococcus, Alistipes*↓; *Lactobacillus*↑ (After prednisone treatment)	The effects of prednisone on gut microbiota were dose-dependent in the treatment of MRL/lpr mice.	(33)

In addition, *Enterococcus gallinarum*, a specific pathogenic bacterium in (NZW × BXSB) F1 lupus mice, was shown to induce intestinal barrier impairments and translocate to the liver to cause autoimmune hepatitis (28). More importantly, *E. gallinarum* was also found in the liver tissues of patients with SLE and autoimmune hepatitis, but not in healthy controls and non-autoimmune hepatitis patients.

Taken together, the above results indicate that intestinal microbiota dysbiosis in an SLE mouse model presents with decreased microbial diversity, increased colonization of harmful bacteria such as *E. gallinarum*, or decreased probiotics. At the same time, impaired intestinal barrier function plays a very important role, which can increase gut microbiota translocation and promote lupus progression.

## 4 Mechanisms of Gut Microbiota Dysbiosis in SLE

Currently, it is unclear whether gut microbiota dysbiosis is the cause or consequence of SLE. Genetic susceptibility is an important factor leading to gut microbiota dysbiosis and autoimmune disease progression in lupus-prone mice (35). However, the data from 1,046 healthy individuals suggested that environmental factors are more important than host genetics in shaping the human gut microbiota (36). In the past decades, the rising incidence of autoimmune diseases has been associated with environmental factors, including a high-salt diet (HSD) (37). Previous studies have shown that HSD could activate DCs and induce the production of pathogenic T Helper 17 (Th17) cells through the p38/MAPK-STAT1 signaling pathway, resulting in gut microbiota dysbiosis, hypertension and autoimmune progression (37–39). Moreover, gut microbiota dysbiosis may induced immune system imbalance and aggravates SLE (40). There are other potential mechanisms underlying for the role of gut microbiota dysbiosis in SLE, which we will elaborate on the following aspects.

### 4.1 Intestinal Barrier Function and Leaky Gut

At present, the pathogenesis of SLE is still not well known, but growing evidence suggests that the impaired intestinal barrier may be one of the essential factors (41). The intestinal mucosa needs the intestinal barrier function to defend against the invasion of foreign antigens, such as food antigens, bacteria, and toxins (7). As previously mentioned, a leaky gut was observed in patients with SLE and in mice. Calprotectin, a calcium-containing protein from neutrophils and macrophages, is a well-recognized biomarker of impaired intestinal barrier function (42). Calprotectin levels were significantly increased in stool samples from patients with SLE, indicating impaired intestinal barrier function (8, 28). At the same time, serum soluble CD14, α1-acid glycoprotein, and lipopolysaccharides (LPSs) levels were increased in patients with SLE, indicating the presence of intestinal bacterial translocation (8).

Interestingly, Thim-Uam et al. (43) used dextran sulfate solution to induce a leaky gut in FcgRIIb^−/−^ lupus mice and pristane-induced lupus mice. They found that the leaky gut aggravated the progression and disease activity of these two murine models of lupus. Leaky gut increases the intestinal translocation of endotoxins or other organic molecules, thereby promoting apoptosis. Most notably, leaky gut promotes the production of anti-dsDNA autoantibodies and immune complex deposition, ultimately leading to lupus exacerbation. Recently, another study indicated that impaired intestinal barrier function is associated with intestinal oxidative stress in MRL/lpr lupus mice (44). This result further complements the mechanism involved in the development of the leaky gut in lupus mice. In addition, impaired gut barrier function and lupus were significantly ameliorated after treatment with antibiotics, probiotics, or dietary interventions in lupus mice (27, 34, 45).

Taken together, these results suggest that impaired intestinal barrier function is associated with SLE disease severity. Both patients with SLE and mice have varying degrees of impaired intestinal barrier function and a leaky gut. Mechanistically, impaired intestinal barrier function allows symbiotic bacteria or their contents to leak out of the intestinal lumen, which may be related to intestinal oxidative stress. Translocated gut bacteria or bacterial components can promote the production of autoantibodies through molecular mimicry. Finally, the deposition of immune complexes aggravates SLE progression.

### 4.2 Molecular Mimicry

Molecular mimicry is another critical condition that leads to the development of autoimmunity (6, 9, 46). Molecular mimicry means that certain structures of a microorganism are similar to the self-structures of the host, which causes an autoimmune response and tissue damage (47). Therefore, certain bacteria with epitope structures similar to self-antigens can stimulate patients with SLE to produce cross-reactive autoantibodies. Zhang et al. (9) found that *Burkholderia* bacterial partial purified antigen and transcriptional regulatory peptide RAGTDEGFG could bind to dsDNA antibodies in sera from patients with SLE (9). These results suggest that the production of anti-dsDNA antibodies in patients with SLE is associated with *Burkholderia* bacterial molecular mimicry. Interestingly, another study found that glycolipids of the mycobacterial cell wall can bind to anti-dsDNA autoantibodies derived from patients with SLE and mice (10). Thus, the production of autoantibodies can result from the molecular mimicry caused by different bacterial infections in SLE. Recently, it has been shown that peptides produced by *Odoribacter splanchnicus* and *Akkermansia muciniphila* bacteria are highly similar to Sm antigen and Fas antigen epitopes (6). More importantly, peptides from these bacteria can activate CD4^+^ T cells or B cells to produce autoantibodies (6). However, these results were limited *in vitro* experiments, *in vivo* experiments need to be designed to confirm these standpoints. In another study, molecular mimicry of commensal or environmental microbes was shown to promote autoantibody production in SLE, which was driven by T cells and HLA-DR restriction (48).

Molecular mimicry has also been associated to the pathogenesis of other autoimmune diseases, such as antiphospholipid syndrome (APS) (49). APS is an autoimmune disease characterized by anti-β2-glycoprotein I (β2GPI) autoantibodies production (50), which can be secondary to SLE (51). On the other hand, the intestinal commensal *Roseburia intestinalis* (*R. int*) mimotope cross-react with β2GPI-reactive memory CD4^+^ Th1 cells and produce anti-*R. int* autoantibodies in patients with APS (49). And oral gavage with *R. int* in BALB/c mice induced anti-human β2GPI autoantibodies and APS-associated autoimmune pathologies (49). Therefore, *R. int* promotes anti-β2GPI autoantibodies production and contributes to APS pathogenesis. In addition, aPL also targeted to β2GPI in SLE (52). Thus, the intestinal commensal *R. int* may be related to the pathogenesis of SLE, but further studies will be required.

In summary, bacterial molecular mimicry is an important factor in the pathogenesis of autoimmune diseases, including SLE and APS. Different bacteria can promote autoantibody production through molecular mimicry. T and B cells are involved in the bacterial molecular mimicry process; however, the precise mechanism remains unclear.

### 4.3 The Pathogenic Role of Bacterial Biofilms

Biofilms are considered to be a membrane in which the bacterial community produces an extracellular matrix and wraps itself (53), which can protect bacteria from the host immune response (54) and enable bacteria to develop drug resistance (55). The main structure of biofilms is amyloid protein rich in β-folding, which is associated with human autoimmune diseases (56–58). Curli fibrils in *Salmonella enterica* serovar Typhimurium (*S.* Typhimurium) amyloid could combined to the DNA in bacterial, and these complexes could promote biofilm formation, also contributing to SLE pathogenesis (57). In pre-lupus NZBxW/F1 mice, curli-DNA complexes activated the innate immune cells such as dendritic cells (DCs) to secrete pathogenic type I interferons (IFNs). NZBxW/F1 lupus-prone mice rapidly developed anti-dsDNA and ANA autoantibodies after intraperitoneal injection of curli-DNA complexes at six weeks of age, whereas injection of BSA did not show the same effect. Most importantly, normal control C57BL/6 mice also developed anti-dsDNA and ANA autoantibodies two weeks after intraperitoneal injection of curli-DNA complexes at six weeks of age. In addition, curli-DNA complexes promoted the proliferation of activated T cells, activated B cells, and inflammatory monocytes. Finally, infection with curli biofilm of *S.* Typhimurium promoted autoantibody production in lupus mice (57). These results suggest that curli-DNA complexes of bacterial biofilms not only promote the production of autoantibodies in lupus-prone mice, but also disrupt self-tolerance in non-autoimmune mice, causing lupus pathogenesis. This has also been shown in another study that curli-bacterial DNA complexes of urinary tract infections in patients with SLE cross-reacted with lupus autoantigens such as dsDNA (59). The above two studies suggest that bacterial proteins that interact with DNA may cause loss of immune tolerance to autoantigens and induce the production of autoantibodies, leading to SLE pathogenesis. Interestingly, a recent study by Fu et al. (58) suggested that DNABII proteins interacting with DNA in biofilms may not directly contribute to anti-dsDNA production but other mechanisms may be involved. Sera from patients with SLE specifically recognize the DNAB II protein-derived HU1 peptide in bacterial biofilms. Anti-HU1 aggravates the progression of lupus nephritis (LN) in patients with SLE and a pristane-induced lupus murine model. Although anti-HU1 antibodies can inhibit biofilm formation by *Staphylococcus aureus*, it is accompanied by cross-reactivity with the autoantigen P4HB on the glomerular cell membrane to induce LN (58).

In conclusion, certain components in bacterial biofilms such as curli and curli-DNA complexes can cross-react with autoantigens and induce the production of autoantibodies, resulting in SLE pathogenesis or disease aggravation.

### 4.4 Intestinal Specific Pathogens Infection

Intestinal infections with specific pathogens have been reported to be associated with the onset and progression of SLE. It is of great significance to study the mechanism of action of these specific pathogens in SLE.

#### 4.4.1 Enterococcus gallinarum

*Enterococcus gallinarum* (*E. gallinarum*) is a human intestinal commensal bacterium that can invade the blood to induce sepsis when the immunity of the organism is low (60). Interestingly, Vieira et al. (28) observed that *E. gallinarum* plays an important role in the pathogenesis of SLE. Pathogenic *E. gallinarum* disrupted intestinal barrier function and promoted Th17 and Tfh cell proliferation in (NZW × BXSB) F1 lupus mice. Subsequently, the damaged intestinal barrier promoted translocation of *E. gallinarum* to mesenteric lymph nodes, mesenteric veins, and liver. At the same time, *E. gallinarum* promoted systemic autoimmunity by inducing ERV gp70 overexpression in the liver (28). Thus, *E. gallinarum* is a pathogenic bacterium that is closely related to the pathogenesis of SLE in (NZW × BXSB) F1 lupus mice. Surprisingly, *E. gallinarum* was detected in liver biopsies from patients with SLE and autoimmune hepatitis, but not in healthy controls and non-autoimmune hepatitis patients. This suggests that *E. gallinarum* of lupus mice were also present in patients with SLE; most importantly, after inoculation with the *E. gallinarum* vaccine, serum autoantibody levels were reduced, the survival time was prolonged, and bacterial translocation was inhibited in (NZW × BXSB) F1 mice (28). Therefore, pathogen-specific therapy can suppress host autoimmune processes without the use of immunosuppressants. More recently, another study showed that *E. gallinarum* is associated with autoimmune responses to autoantibodies such as anti-Ribosomal P, anti-dsDNA, and anti-Sm in patients with SLE (61). This study further confirmed that *E. gallinarum* is a specific pathogen of SLE-susceptible individuals. However, this study did not prove that whether *E. gallinarum* acts as the same role in other lupus mouse models and patients with SLE.

Taken together, the pathogenic bacteria *E. gallinarum* can be translocated into systemic organs by disrupting the intestinal barrier, which leads to SLE pathogenesis. Translocated *E. gallinarum* promotes Th17 and Tfh cell proliferation and autoantibody production. At the same time, *E. gallinarum* may also directly induce autoantigens, ERV proteins, and other substances to promote autoimmune processes.

#### 4.4.2 Ruminococcus gnavus

As previously mentioned, *Ruminococcus gnavus* (*R. gnavus*) plays an important role in SLE (6, 8). Studies have shown that *R. gnavus* expresses a B-cell superantigen that stimulates the gut of mice to produce large amounts of plasma cells that secrete IgA antibodies (62) These IgA antibodies recognize and highly encapsulate *R. gnavus*, which may be associated with intestinal colonization of *R. gnavus*. Furthermore, *R. gnavus* can produce a glucorhamnan inflammatory polysaccharide that promotes dendritic cells to secrete the inflammatory factor Tumor necrosis factor-α (TNF-α) *via* toll-like receptor4 (TLR4) (63). A recent study has shown that some isolated strains of *R. gnavus* could produce capsular polysaccharides that promote the immune tolerance of *R. gnavus*. However, *R. gnavus* isolates without capsular polysaccharide produced a strong pro-inflammatory response and increased intestinal inflammatory indicators in sterile mice (64).

Interestingly, Azzouz et al. found that sIgA-coated *R. gnavus* increased in stool samples from patients with SLE, and the proliferation of *R. gnavus* was proportional to SLE disease activity (8). Thus, aberrant superantigen expression of *R. gnavus* may facilitate intestinal colonization of *R. gnavus*, thereby aggravating SLE progression. In addition, *R. gnavus* can disrupt intestinal barrier function, resulting in increased levels of calprotectin in stool samples and lipopolysaccharides (LPSs) in sera. Subsequently, the impaired intestinal barrier function exposes the intestinal commensal *R. gnavus* antigen, leading to mimicry of the molecule to produce anti-dsDNA autoantibodies, aggravating lupus (8).

In summary, *R. gnavus* may affect disease progression in SLE, but the causal relationship remains unresolved.

### 4.5 Gender Bias

Generally, SLE shows a strong female bias with a male-to-female ratio of 9:1 (65). In fact, there was also a gender bias in the intestinal microbiota in SLE. For example, over-colonization of *Lachnospiraceae* in the intestinal tract of female MRL/lpr lupus mice was associated with early onset or exacerbation of lupus, but not in male mice (26). Another study showed an increase in *Lachnospiraceae* and exacerbation of lupus in the gut microbiota of MRL/lpr mice after administering a phytoestrogen-supplemented diet (66). These results suggest that estrogen may account for gender bias in gut microbiota dysbiosis in SLE, but the underlying mechanism remains to be clarified. Moreover, estradiol exacerbates SLE disease severity by promoting type I interferon responses and IgG autoantibody production from B cells (67, 68). Abnormal modification of steroid receptors in T cells may alter the expression of estrogen receptor (ERα), thereby promoting the effect of estrogen (65). In contrast, testosterone is generally considered to be a beneficial sex hormone that inhibits B-cell activation and autoantibody production to alleviate LN (69). On the other hand, Mu et al. found that *Lactobacillus* treatment ameliorated lupus nephritis, increased IL-10, and decreased luteinizing hormone in female and emasculated male MRL/lpr mice, but not in intact male mice (11). These results suggest that *Lactobacillus* treatment ameliorates LN in MRL/lpr mice in a sex hormone-dependent manner. In addition, antibiotic treatment has been shown to inhibit SLE progression in lupus-prone (SWR × NZB) F1 female mice, but not in male mice. Orchiectomy alters the composition of the gut microbiota and promotes autoimmune progression in male mice (70).

In conclusion, estrogen can alter gut microbiota and promote type I interferon response and autoantibody production to aggravate SLE progression; conversely, androgen plays a protective role.

### 4.6 Intestinal Epithelial Cells Autophagy

At present, the relationship between autophagy and intestinal bacteria in SLE has not been reported. However, in another autoimmune disease, inflammatory bowel disease (IBD), autophagy is crucial for the homeostasis of intestinal bacteria and intestinal barrier function. On the one hand, autophagy may be beneficial to gut barrier function. The autophagic protein Atg16L1 prevents necrotizing apoptosis mediated by TNF-α in intestinal epithelial cells (IECs) by promoting mitochondrial homeostasis (71). Autophagy can also reduce epithelial permeability by inducing lysosomal degradation of the pore-forming tight junction protein claudin-2, thus enhancing intestinal barrier function (72). On the other hand, IECs autophagy plays a crucial role in regulating the diversity and composition of the gut microbiota. For example, the estrogen-associated receptor alpha (ESRRA) protects the host from mitochondrial dysfunction by activating autophagy and maintaining intestinal microbiota homeostasis, thereby attenuating intestinal inflammation (73). IECs-specific knockout of autophagy-associated gene 5 (Atg5) resulted in significant changes and decreased diversity of gut microbiota in mice. In Atg5-deficient mice, the abundance of inflammation-inhibiting *Akkermansia muciniphila* decreased, but the abundance of pro-inflammatory *Candidatus* Athromitus and potentially pathogenic *Pasteurellaceae* increased (74). In addition, fecal microbiota transplantation could increase the expression of LC3B and Atg7 to activate intestinal mucosal autophagy, thereby improving intestinal barrier function in piglets (75). These studies suggest that autophagy of host intestinal mucosal cells may affect the gut microbiota to ameliorate intestinal injury.

The previous discussion indicates that dysregulation of gut microbiota and impaired intestinal barrier function can lead to aggravated SLE progression. IECs autophagy contributes to the maintenance of gut microbiota homeostasis and intestinal barrier function. A review by Bhattacharya et al. (76) indicated that exploring the mechanism of the interaction between autophagy and gut microbiota is beneficial for the study of autoimmune diseases. Therefore, we hypothesized that IECs autophagy is closely related to the dysregulation of gut microbiota in SLE and affects the progression of SLE. However, further studies are required to confirm our findings.

### 4.7 Extracellular Vesicle and miRNA

Extracellular vesicles (EVs) are a group of membrane-enclosed nanoscale vesicles that carry various RNA, DNA, proteins, and lipids and transmit information between cells (77). Exosomes are EVs ranging in diameter from approximately 40 to 160 nm that carry miRNAs and other non-coding RNAs with the potential for diagnosis and treatment of diseases (78). miRNAs are single-stranded non-coding RNA molecules of approximately 22 nucleotides in length that play important roles in regulating gene expression and biological function (79). In recent years, studies have shown that EV-derived miRNA expression is related to gut microbiota and intestinal barrier function (80–82). Mice deficient in IECs miRNA showed intestinal dysbiosis and exacerbation of colitis, which ameliorated after transplantation with fecal EV-derived miRNA from wild-type mice (80). This study suggests that fecal EV-derived miRNAs can regulate the gut microbiota and ameliorate the progression of intestinal inflammation. Another study found that EV-derived miRNAs of dietary ginger can induce IL-22 production to improve intestinal barrier function and thus ameliorate intestinal inflammation (83). A recent study showed that exosome miR-181a derived from MSCs alleviated colitis by improving gut microbiota imbalance and intestinal barrier function and reducing pro-inflammatory factor secretion (82). Taken together, the above results suggest that some EV-derived miRNAs in the intestinal tract may inhibit the progression of SLE by improving gut microbiota homeostasis and intestinal barrier function. More studies are required to confirm this hypothesis.

## 5 Potential Therapy for SLE: Modulating Gut Microbiota

At present, the study of intestinal bacteria intervention in the treatment of SLE is still in its infancy, but it can learn from other dysbacteriosis-associated diseases and predict future regimens in the treatment of SLE. As described in a recent review (40), probiotics/prebiotic therapy are currently practical approaches for ameliorating intestinal dysbacteriosis to treat SLE. Probiotics and prebiotics can induce differentiation of Treg cells, improve Th17/Th1 imbalance, and reduce the production of autoantibodies, thereby reducing the severity of lupus (40). Nevertheless, the efficacy of probiotics/prebiotic in the treatment of SLE remains unclear and has not been confirmed by clinical trials. There are differences in phenotypic manifestations caused by gut microbiota in SLE. To illustrate, intestinal commensal *E. gallinarum* can translocation to the liver and cause autoimmune hepatitis in patients with SLE (28). *R. gnavus* could increase serum anti-dsDNA antibody and LPS levels (8). The curli-DNA complex of biofilms containing *S.* Typhimurium promoted lupus progression. These differences may influence the approaches to targeting gut microbiota for SLE. These differences may have an impact on choosing the most appropriate modulation method of gut microbiota. Next, we discuss several options for the intervention of gut microbiota in the treatment of SLE.

### 5.1 Dietary Intervention

Dietary intervention may regulate the imbalance of gut microbiota, and thus ameliorate SLE progression. The alteration of the pH value of drinking water could beneficially influence on gut microbiota composition and disease progression in SWR×NZB F1(SNF1) lupus mice (84). Dietary retinoic acids supplementation could upregulate lactobacilli and ameliorate lupus in MRL/lpr mice (26). However, the efficacy of dietary retinoic acids in SLE treatment remains controversial (45) and still needs further study.

Also, the high-salt diet plays an important role in the pathogenesis of gut microbiota dysbiosis in autoimmune diseases. Interestingly, a recent randomized controlled trial demonstrated that a low-salt diet increased circulating SCFAs and decreased blood pressures by affecting the gut microbiota in humans (85). Therefore, reducing dietary salt intake or targeting salt-sensitive associated protein may be a new therapeutic strategy for SLE treatment. But this strategy still needs to be confirmed by further studies.

In addition, celiac disease (CeD) is an autoimmune enteropathy that is proposed to be associated with SLE (86, 87). An analysis of 29,000 patients with biopsy-confirmed CeD found that patients with CeD had a three-fold increased risk of developing SLE compared with healthy controls (86). In contrast, a large case-control study involving 5018 patients with SLE reported a significantly higher prevalence of CeD in patients with SLE compared with matched controls (87). And gluten, the major protein of wheat grains, is one of the factors contributed to the coexistence of SLE and CeD (87). The gliadin polypeptide of gluten increased intestinal permeability and activated CD4^+^ T cells resulted in CeD (88, 89). Therefore, gluten may be one of the causes of impaired intestinal barrier function in patients with SLE. Currently, the gold standard treatment for CeD is a strict and life-long gluten-free diet (GFD) (90). However, the implementation of GFD is limited by high cost, decreased quality of life of patients and complex pathogenesis (90). GFD may contribute to improve gut barrier function but still requires additional study.

Altogether, dietary intervention may be an important and new therapy in SLE.

### 5.2 Oral Antibiotic Therapy

In recent years, many studies have attempted to use antibiotics to treat lupus mice. For example, treatment with broad-spectrum antibiotics or vancomycin after onset in lupus MRL/lpr mice removes harmful bacteria from the gut, enriches probiotics, and restores gut barrier function, thereby ameliorating lupus (27). Moreover, antibiotic treatment alleviates Treg/Th17 imbalance in lupus mice (27) and inhibits the high blood pressure caused by Th17 cell infiltration (32). Vieira et al. (28) found that vancomycin treatment of NZB/WF1 lupus mice cleared *E. gallinarum*, a specific pathogen in the intestine, improved intestinal barrier function, and delayed lupus progression. However, another study showed that treatment with antibiotics has no significant effect on both the gut microbiota and SLE progression in NZB/WF1 lupus mice, the mechanism of which is unclear (91). Similarly, Zhang et al. showed that antibiotic treatment exacerbated the disease in MRL/lpr mice, possibly due to a short course and insufficient dose of antibiotics before lupus onset (31). Alternatively, another study found that vancomycin treatment ameliorated lupus symptoms in common MRL/lpr mice, but aggravated lupus in pregnant and postpartum (PP) mice. Mechanistically, vancomycin treatment aggravates LN in PP mice by downregulating the expression of Treg cells through inhibition of IDO and upregulation of IFN-γ (29).

In conclusion, the antibiotic therapy regimen for SLE is controversial. In general, antibiotic treatment decreases pathogenic bacteria, enriches probiotics, and ameliorates intestinal leakage in lupus mice, thereby inhibiting lupus progression. However, antibiotics may also exacerbate lupus severity in premorbid and pregnant or lactating mice. Moreover, there are some limitations in the routine use of antibiotics to treat patients with SLE. Because antibiotic treatment may inhibit the therapeutic effect of prednisone on lupus in MRL/lpr mice (31), while prednisone is a common drug for patients with SLE in clinical practice. Furthermore, antibiotic abuse may lead to drug-resistant bacterial infection (92), which is an important cause of death in patients with SLE (93). Therefore, the use of antibiotics in the treatment of SLE needs to be further studied to specifically remove pathogenic bacteria without causing gut microbiota disorders as much as possible.

### 5.3 Fecal Microbiota Transplantation

Fecal microbiota transplantation (FMT) is defined as the transplantation of bacteria from the feces of healthy donors into the patient’s intestine to restore microecology homeostasis and thus treat diseases associated with gut microbiota imbalance (94). In 2013, FMT was included in the official therapeutic guidelines for *Clostridium difficile* infection (CDI) (95). In recent years, studies have shown that FMT is effective in the treatment of SLE mouse models (31, 33, 84). An acidic water diet can restore the balance of gut microbiota in lupus mice, and that this repaired gut microbiota can be used for FMT to treat control lupus mice [25703185]. In addition, short-term antibiotic treatment of early-stage MRL/lpr lupus mice promoted SLE progression, and the disease severity in these mice was reduced after FMT treatment in the following week. However, short-term premorbid antibiotic treatment or FMT could inhibit the therapeutic effect of prednisone on lupus in MRL/lpr mice aged 9 to 13 weeks (31). This study suggests that performing FMT early in the onset of lupus suppresses the progression of lupus, but, at the same time, affects the therapeutic effect of glucocorticoid therapy. If patients with SLE are routinely treated with glucocorticoids, treatment with FMT should be carefully considered. More recently, a study found that untreated lupus MRL/lpr mice transplanted with fecal microbiota from prednisone-treated mice experienced lupus attenuated without the side effects of prednisone-treated mice (33). These results suggest that FMT may be an effective SLE therapy to avoid adverse glucocorticoid reactions. The effect of the interaction between FMT and glucocorticoid therapy on the progression of SLE requires further study. FMT clinical trials have been studied for other autoimmune diseases, such as ulcer colitis and type 1 diabetes, and some efficacy has been achieved (96, 97). Therefore, clinical trials of FMT in patients with SLE are promising, but further studies are needed.

A recent article reported that a patient succumbed to infection due to drug-resistant *E. coli* bacteria in donor stool samples (98). Therefore, donor screening must be improved to prevent transmission of microorganisms leading to infectious events. In conclusion, the benefits and risks of FMT in the treatment of SLE need to be assessed, and how to apply it in clinical practice still needs further study.

### 5.4 Glucocorticoid Therapy

Glucocorticoids (GCs) are steroids that can bind and activate the cytosolic glucocorticoid receptors (GRs) to exert an anti-inflammatory effect (99). GCs have become one of the main traditional drugs for SLE due to their rapid and potent anti-inflammatory effects, low cost, and easy availability. Moreover, long-term high-dose GC treatment regimens are accompanied by an increase in side effects and infections (100). Enhancing the efficacy of GCs and reducing their side effects in patients with SLE is a challenge. In recent years, studies have shown that the efficacy of GCs in the treatment of SLE is related to changes in the gut microbiota. For example, NZB/W F1 mice treated with dexamethasone had increased diversity of intestinal bacteria and a significant reduction in a certain *Lactobacillus* species associated with lupus progression (101). In another study, prednisone treatment caused alterations in the gut microbiota, including a decrease in *Mucispirillum* and an increase in *Anaerostipes*, which were inversely associated with disease activity in SLE. Bromofuranone did not alleviate lupus but enhanced the efficacy of prednisone in the treatment of SLE (30). As previously mentioned, Wang et al. demonstrated that prednisone ameliorates gut microbiota dysbiosis in SLE mouse models, and FMT treatment of SLE may prevent glucocorticoid adverse reactions (33). Moreover, GCs treatment restored the gut *Firmicutes/Bacteroidetes* ratio and increased the abundance of probiotics such as *Lactobacillus* and *Bifidobacterium* in patients with SLE (19).

All those studies suggest that intestinal dysbacteriosis may be a target for GCs in the treatment of SLE, but the mechanism remains unclear. A comparative study indicated that an increased levels of *Lactobacillus* in patients with SLE under GCs treatment (19). Moreover, *Lactobacillus* contributes to the alleviation of lupus severity by upregulating Foxp3^+^ regulatory T (Treg) cells (102). Treg cells are indispensable GC target cells *in vivo* (103). And GCs directly act on GRs in Treg cells and regulate miR-342-3p dependent metabolic programming to exert therapeutic effects (103). Therefore, *Lactobacillus* may affect the therapeutic efficacy of GCs by promoting the proliferation of Treg cells.

Overall, intestinal dysbacteriosis is one of the targets of GCs in the treatment of SLE. Certain drugs such as bromofuranone are associated with enhancing the therapeutic effects of GCs, and FMT may be an effective treatment regimen to reduce the side effects of GCs. Certain intestinal bacteria such as *Lactobacillus* may affected the therapeutic effect of GCs by regulating Treg cells. However, the specific mechanism by which GCs modulate intestinal bacteria in the treatment of SLE needs to be elucidated in future studies.

### 5.5 Regulate IECs Autophagy and EV-Derived miRNA Therapy

Autophagy is crucial for maintaining the homeostasis of gut microbiota and intestinal barrier function (71, 72). Therefore, regulating IECs autophagy may help improve the gut microbiota balance for the treatment of SLE. First, some drugs can improve gut microbiota composition and intestinal barrier function by promoting IECs autophagy, thus reducing intestinal inflammation and inhibiting autoimmune (104–106). For example, rapamycin can inhibit the progression of multiple sclerosis by promoting IECs autophagy and restoring intestinal microbiota balance (107). Galangin increases the expression of autophagy-related proteins and promotes the formation of colonic autophagy, increases the richness of intestinal probiotics, and reduces intestinal inflammation (108). Second, FMT can increase the expression of intestinal mucosal autophagy-related proteins and reduce intestinal permeability in piglets (75).

In contrast, EV-derived miRNAs may treat SLE by modulating the gut microbiota. A recent review reported that food-derived miRNAs could regulate the composition of gut microbiota and enhance intestinal barrier function, which is beneficial to human health (109). For instance, dietary ginger-derived miRNAs can induce IL-22 production to improve intestinal barrier function and thus ameliorate intestinal inflammation (83). Furthermore, MSC-derived exosome miR-181a can alleviate colitis by improving gut microbiota imbalance and intestinal barrier function and reducing pro-inflammatory factor secretion (82). Mice deficient in IECs miRNA showed intestinal dysbiosis and exacerbation of colitis, which ameliorated after transplantation with fecal EV-derived miRNA from wild-type mice (80).

In summary, IECs autophagy and EV-derived miRNAs can restore gut microbiota balance and intestinal barrier function, thereby inhibiting autoimmune-related intestinal inflammation. Therefore, we believe that regulating autophagy and EV-derived miRNAs is a promising therapeutic option for SLE.

### 5.6 Mesenchymal Stem Cell Therapy

MSCs are stromal cells with self-renewal and multi-lineage differentiation potential that can be obtained from tissues such as bone marrow (110). With low immunogenicity and strong immunomodulatory effects (111), MSCs can be used to treat SLE (112, 113). Allogeneic MSC transplantation ameliorates clinical symptoms, decreases SLEDAI score, and ameliorates LN in patients with refractory SLE (113, 114). Moreover, studies have shown that MSCs can regulate gut microbiota, increase insulin-like growth factor-1 (IGF-1), promote intestinal healing, and ameliorate the mouse model of IBD (115, 116). In contrast, miRNA-181a of MSC-derived exosomes can attenuate intestinal inflammation in mouse models of colitis by improving the composition of gut microbiota and restoring barrier function. These studies suggest that MSCs or MSC-derived exosomes can improve gut microbiota dysbiosis and intestinal barrier function and ameliorate intestinal inflammation (82).

Recently, it has been shown that human umbilical mesenchymal stem cells (hUC-MSCs) treat rheumatoid arthritis (RA) by regulating the interaction between gut microbiota and host immunity through the aryl hydrocarbon receptor (AhR) (117). Thus, we hypothesized that MSCs can inhibit SLE progression by ameliorating gut microbiota dysbiosis and intestinal barrier function. However, the specific mechanism of action of MSCs in the treatment of SLE is unknown, and the therapeutic effects of clinical trials remain controversial. In a clinical trial, hUC-MSCs did not have a positive therapeutic effect in patients with severe LN compared with placebo control (118). Meanwhile, a review suggested that the immunomodulatory effects of MSCs depend on the inflammation status, and that MSCs can both suppress and promote immune responses (119). Moreover, gut microbiota dysbiosis may inhibit the therapeutic effect of MSCs. For example, chronic hypoxia has been found to lead to intestinal dysbiosis and promote senescence of bone marrow MSCs (120). Another study demonstrated that intestinal dysbacteriosis might inhibit the therapeutic effect of MSCs in diabetic mice, while modulation of intestinal bacteria may help to enhance the therapeutic effect of MSC transplantation (121). These results suggest that intestinal bacteria can affect the immunomodulatory effects of MSCs, which may be one of the reasons for the poor efficacy of some MSCs in the treatment of SLE. In addition, Ocansey’s review (122) suggested that there would be a higher clinical remission rate in patients with IBD treated with the combined MSC-FMT transplantation approach compared with MSC transplantation alone or FMT transplantation. Similarly, we believe that the combined MSC-FMT transplantation approach will have a better therapeutic effect in the treatment of SLE.

Taken together, the study of MSCs in the treatment of SLE has fallen into a bottleneck, and gut microbiota will be a very promising direction for future research.

### 5.7 Vaccination

To prevent infection, the EULAR guidelines recommend vaccinations such as pneumococcal vaccines (PCV13) for patients with SLE during inactive periods (123). Vaccination of MRL/lpr mice with PCV13 ameliorated lupus severity (124). As previously described, SLE mouse models may suffer from infection with specific intestinal pathogens, such as *E. gallinarum* and *R. gnavus* (8, 28). The development of vaccines against these specific pathogenic bacteria could contribute to the treatment of SLE. Accordingly, Vieira et al. (28) demonstrated that after inoculation of *E. gallinarum* vaccine in (NZW × BXSB) F1 lupus mice, intestinal barrier function was restored and SLE was alleviated. This study shows that specific targeted therapy for intestinal pathogens can inhibit host autoimmune progression independent of other drugs. Importantly, *E. gallinarum* was also detected in the gut and liver of patients with SLE (28). This illustrates that *the E. gallinarum* vaccine is very promising for the treatment of patients with SLE, but further studies are still needed.

At present, research on intestinal microbiota vaccines is still in the preliminary stage, but there is no doubt that targeted vaccine therapy of specific intestinal pathogens is a very promising treatment for SLE.

## 6 Future Perspectives

Gut microbiota dysbiosis is closely related to the occurrence and development of SLE. The interaction of factors such as impaired intestinal barrier function, molecular mimicry, biofilms, specific pathogens, and sex hormones can disrupt gut microbiota balance and aggravate SLE. In addition, we suggest that IECs autophagy and EV-derived miRNAs may also affect progression in SLE by regulating the gut microbiota.

Traditionally, it is difficult to treat SLE due to its heterogeneity and complex pathogenesis, while targeting intestinal bacteria may be a breakthrough. In previous studies, probiotics or prebiotic modulation of intestinal bacteria have shown some efficacy in the treatment of SLE, but it is still controversial and has not been confirmed in clinical trials. Dietary interventions such as oral retinoic acids, low-salt diets and gluten-free diets may be beneficial in the treatment of SLE, but further research is needed. Oral antibiotics have some efficacy but may lead to more severe intestinal dysbacteriosis or the development of drug-resistant bacteria. Vaccination against gut pathogenic bacteria suppresses lupus progression in (NZW × BXSB) F1 mice without antibiotic-related side effects, but it is not yet available to treat all lupus mouse and patients. Notably, FMT significantly ameliorates disease in lupus mice by restoring the intestinal bacterial balance and intestinal barrier function. Clinical trials of FMT in patients with SLE are promising; however, donor stool screening must be improved to prevent infectious events.

We also propose new insights into the regulation of gut microbiota in SLE, including GCs, autophagy, EV-derived miRNAs, and MSC therapy. First, GCs, which are commonly used in SLE, can ameliorate intestinal dysbacteriosis but have side effects. The regulation of gut microbiota may help enhance the efficacy of GCs in the treatment of SLE and prevent side effects. Second, regulating autophagy and EV-derived miRNAs may treat SLE by regulating the gut microbiota. Finally, promising results have been achieved for the use of MSCs in patients with refractory SLE in current clinical trials. However, disturbed gut microbiota may inhibit the therapeutic effects of MSCs. In contrast, MSCs can ameliorate intestinal dysbacteriosis, restore intestinal barrier function, and inhibit autoimmune progression. In addition, MSC-derived EVs could ameliorate RA in rats by modulating the gut microbiota. Therefore, the gut microbiota may be a target for MSCs in the treatment of SLE. Moreover, the combination of MSC-FMT transplantation has the potential to enhance the effect of MSCs in the treatment of SLE. Therefore, MSC regulation of gut microbiota for the treatment of SLE is a promising direction for future study.

Here, we summarize novel insights into the mechanisms of microbiota dysbiosis in SLE and provide promising therapeutic strategies, which may help improve our understanding of the pathogenesis of SLE and provide novel therapies for SLE.

## Author Contributions

QRP and FG wrote the manuscript and designed the figures. YH, AL, SC, JC, and H-FL revised the manuscript. All authors contributed to the article and approved the submitted version.

## Funding

This study was supported by National Natural Science Foundation of China (no.82070757), the Project of “Dengfeng Plan” and Department of established positions for the Zhujiang Scholar from Guangdong Medical University, and Guangdong Basic and Applied Basic Research Foundation (no.2019A1515012203), the Zhanjiang City Program for Tackling Key Problems in Science and Technology (no. 2019B01179, no. 2017A01010).

## Conflict of Interest

The authors declare that the research was conducted in the absence of any commercial or financial relationships that could be construed as a potential conflict of interest.

## Publisher’s Note

All claims expressed in this article are solely those of the authors and do not necessarily represent those of their affiliated organizations, or those of the publisher, the editors and the reviewers. Any product that may be evaluated in this article, or claim that may be made by its manufacturer, is not guaranteed or endorsed by the publisher.
